# Challenges of E-Learning: Behavioral Intention of Academicians to Use E-Learning during COVID-19 Crisis

**DOI:** 10.3390/jpm13030555

**Published:** 2023-03-20

**Authors:** Mohammad Jamal Khan, Lingala Kalyan Viswanath Reddy, Javed Khan, Bayapa Reddy Narapureddy, Sunil Kumar Vaddamanu, Fahad Hussain Alhamoudi, Rajesh Vyas, Vishwanath Gurumurthy, Abdelrhman Ahmed Galaleldin Altijani, Saurabh Chaturvedi

**Affiliations:** 1 Department of Public Health, College of Health Sciences, Saudi Electronic University, Riyadh 13316, Saudi Arabia; moha.khan@seu.edu.sa (M.J.K.); j.khan@seu.edu.sa (J.K.); 2 Department of Public Health, College of Health Sciences, Saudi Electronic University, Abha 61421, Saudi Arabia; 3 Department of Public Health, College of Applied Medical Sciences, Khamis Mushayt, King Khalid University, Abha 61421, Saudi Arabia; bayapreddy916@gmail.com (B.R.N.); aaltijani@kku.edu.sa (A.A.G.A.); 4 Department of Dental Technology, College of Applied Medical Sciences, King Khalid University, Abha 61421, Saudi Arabia; snu@kku.edu.sa (S.K.V.); fhali@kku.edu.sa (F.H.A.); rvyas@kku.edu.sa (R.V.); vishwa.stem@gmail.com (V.G.); 5 Department of Prosthetic Dentistry, College of Dentistry, King Khalid University, Abha 61421, Saudi Arabia

**Keywords:** attitude, e-learning, behaviour, technology acceptance model, COVID-19

## Abstract

The COVID-19 crisis demanded that all educational activities should be performed virtually to follow social distancing guidelines. Therefore, there was a need to perform a research study to assess the effects of external factors on the perceived usefulness, ease of use of e-learning, and the further effect of these perceptions on attitude and intent to use e-learning by using the technology acceptance model (TAM) among academicians at higher education institutions in the Kingdom of Saudi Arabia. Methods: A cross-sectional study was conducted, and data were collected from 263 academicians across Saudi Arabia through an online survey questionnaire using a non-probability purposive sampling technique and analyzed and tested using the SPSS and Smart PLS software. Results: This study found that self-efficacy was positively associated with perceived usefulness at β = 0.143 and *p* < 0.05, but it had no association with perceived ease of use at β = 0.057 at *p* > 0.05. System accessibility had a significant and positive relationship with perceived usefulness and perceived ease of use at β = 0.283, β = 0.247, and *p* < 0.01, respectively. Self-efficacy had a positive effect on perceived usefulness, whereas the subjective norm had no relationship with perceived usefulness and perceived ease of use at β = −0.065 and β = −0.012 at *p* > 0.05, respectively. Perceived ease of use and perceived usefulness were positively related to attitude towards use, which has a significant influence on intention to use e-learning. Conclusion: Perceived ease of application is the most significant factor (β = 0.556) in developing the attitude among academicians to practice e-learning, followed by perceived usefulness (β = 0.262). Moreover, it can be concluded that system accessibility has a stronger influence on developing perception among academicians about the expediency and ease of application of e-learning than self-efficacy.

## 1. Introduction

The novel COVID-19 pandemic has changed the routine courses of action in every aspect of day-to-day work, and the education system was no exception to it. The pandemic shut down every educational institution all over the world and forced institutes of higher learning to abruptly shift their physical learning practices to e-learning [1,2]. At the outbreak of the disease, almost every institution of higher learning across the globe shifted their teaching activity from classroom teaching to virtual teaching, including in Saudi Arabia, to curb the spread of the COVID-19 virus obligation to follow social distancing. Shifting to virtual classes and adopting e-learning could be relatively easy for the students as the current generation of students were born in the information technology (IT) era. However, adopting e-learning might be difficult for academicians as many of them are not familiar with e-learning, as institutions of higher education in developing regions are lagging in the implementation of e-learning [3,4]. E-learning adoption is a steady process that gradually develops perceptions and attitudes among prospective users and ultimately leads to the intended use of technology. However, the rapid spread of COVID-19 denied academicians a chance to follow the process of adopting e-learning. Thus, there is a need to stud the behavioral Intention of Academicians to Use e-Learning during the COVID-19 Crisis.

Information and communication technology (ICT) is being used as an instrument for teachers and students to determine learning concepts, resolve problems, and offer answers to the problems in the learning course [5]. The components of e-learning encompass content delivery in diverse formats, mentors’ squads, students, curriculum designers, etc. [6]. E-learning stabilizes the teaching procedure by covering distances and reaching out to the masses [7]. The primary advantage of e-learning is that it integrates altogether various instructive events that are accepted by groups or individuals working offline or online, synchronously or asynchronously via stand-alone or networked computers and other electronic gadgets [8]. The e-learning concept focuses on not just online learning but also embedded virtual simulated learning, distributed learning (in real time on or off campus), and networked or web-based learning [9]. The primary purpose of e-learning is to minimize the time needed for the pupil to learn by providing specialized latest available information [10]. To successfully integrate e-learning into regular teaching, the curriculum developers need to restructure courses according to student-centered ability [11]. Moreover, the faculty and end users must possess specific skills for using e-learning tools [12].

A thorough literature review found that previous studies have made sincere attempts to study the acceptance of e-learning from the perspective of higher education institutions by using the technology acceptance model (TAM) [13,14,15,16,17,18]. However, these studies mainly explored the perceptions, intentions, and attitudes of students on the acceptance of e-learning. The literature on TAM largely lacks in exploring the perception, attitude, and intention among academicians at institutes of higher learning on acceptance of e-learning. Further, most recent literature on the use of TAM in the acceptance of e-learning is lacking in understanding the perception, attitude, and intention of the use of e-learning among academicians when the method of teaching was abruptly changed from the traditional classroom to virtual, distributed learning due to COVID-19 pandemic. Most recent studies on e-learning during the COVID-19 predicament have explored the significant factors, opportunities, and challenges of e-learning [19,20,21]. However, these studies did not use the TAM model to understand the impact of external factors on the perceived ease of use and perceived usefulness of e-learning that ultimately develops the intention and attitude towards the application of e-learning among academicians.

This research study aimed to explore the effects of external factors on the perceived ease of use and perceived usefulness of e-learning and the further effect of these perceptions on attitude and intention to use e-learning by academicians at higher education institutions in Saudi Arabia using the technology acceptance model (TAM). The uniqueness of this study lies in the fact that it was conducted during the crisis period, and unlike previous studies, which were based on students, this study helped in understanding the perception, attitude, and intention of the use of e-learning among academicians when the method of teaching was abruptly changed from the traditional classroom to virtual, distributed learning due to COVID-19 pandemic. The study is of great significance as it effectively bridges the gap in the literature in terms of applying TAM by academicians at higher education institutions during the COVID-19 Emergency. There is a scarcity of the literature that explored the perceptions, attitudes, and intentions of the application of e-learning among academicians during the crisis. This study would be of help in filling this gap as through the survey and by assessing the results, we will be able to assess the said aim of the study and would recommend the significance of e-learning in the future.

The basic research questions on which this study was based were to evaluate the influence of external variables (system accessibility, self-efficacy, and subjective norms) on perceived ease of use and perceived usefulness of e-learning among academicians, assess the influence of perceived ease of use and perceived usefulness on attitude to apply e-learning among academicians, and analyze the impact of attitude towards use on purpose to use e-learning among academicians.

### 1.1. Literature Review

#### 1.1.1. Technology Acceptance Model (TAM)

The TAM is one of the best forefront rescuers to comprehend technology-related acceptance for e-learners in various instances. The TAM developed earlier describes the individual factors that influence the perception and attitude of individuals toward adopting new technology. The TAM is one of the e-learning acceptance models used to appreciate and boost the attitude of students and faculty. This model also explains that when individual perceptions such as perceived ease of use (PEU) and perceived usefulness (PU) about new technology are high, individuals will possess a constructive attitude toward new technology that creates an intention to use the technology. Nguyen et al. stated that participants’ perspectives of technology acceptance in palliative care were largely dependent on their potential to help address major challenges in the field without imposing a significant burden on providers and patients. The TAM theory points out that an individual’s intent to use and usage behavior of technology is based on the convenience and usefulness of technology as per the individual’s perception [22,23,24]. This theory comprises variables such as PEU, PU, and attitudes towards acceptance of new technology by the users [25] and focuses on the end user’s perspective on new technologies for determining the behavioral intention factors [26].

#### 1.1.2. External Factors (Self-Efficacy, System Accessibility, and Subjective Norm)

Self-efficacy has an essential role in developing the motivation and behavior of individuals, referring to an individual’s experience of her/his ability to accomplish a task or involve in an activity [27,28]. According to Bandura [27], “self-efficacy means beliefs one’s own capabilities to cognitive resources, self-motivation, and sequence of action needed to meet demands under given situation”. System accessibility refers to the issue of the delivery system. It is defined as the quality of access to the delivery system, which explains the varied user behavior in the situation of the existence of alternatives. In a previous study about mobile apps and telemedicine, it was stated that is a strong forecaster of the consumer’s perception of health apps in smartphones towards telemedicine. System accessibility is multi-dimensional, which includes both physical access to the device and information system as well as the capacity to use the system naturally [29,30].

Subjective norm is defined as the specific behavior of an individual towards the people whom he/she perceives are most important to that individual [31]. The most significant individuals within an organizational setting are typically peer groups and managers [32]. The degree to which a person considers how others may affect his or her behavior is, thus, the subject norm. Subjective norms are a significant part of the TAM’s explanation of people’s attitudes toward and intentions for adopting new technologies. According to Venkatesh and Davis [33], a person will consider a new system beneficial if their coworker finds it useful. A person can act in a certain way while being influenced by a coworker without liking the action or the results.

#### 1.1.3. Perceived Ease of Use (PEU) and Perceived Usefulness (PU)

Perceived usefulness refers to an individual’s belief that improved job performance depends on the extent to which technology is applied [34]. The extent to which a person believes that a certain technology will be used naturally without much effort is referred to as perceived ease of use [22]. Perceived usefulness an individual believes that her/his job performance is enhanced proportionately to the extent of using technology. At the same time, perceived ease of use explains the amount of effort an individual makes in using the technology. Suppose a person believes that there is too much hard work to enhance performance by using technology, and efforts put into using technology outweigh the benefits. In that case, the person will not use the technology. According to Karahanna and Straub [35], the usage of technology is influenced by the perceived ease of use of technology.

The term usefulness defines the quality of something to be useful. In the organizational context usefulness of new technology can be measured by the level of performance of the employees. If performance is high, the technology is useful. As high performance leads to reinforcement, such as increments, promotions, and rewards, employees use the new technology effectively to receive reinforcement. The term ease defines the absence of difficulty or great efforts. The usage of new technology needs effort; however, the effort is a resource that is limited. An individual can only apply limited efforts to perform an action. To rephrase it, it can be argued that a technology that can be easily used will consider useful.

#### 1.1.4. Attitude (Towards Use)

Attitude is the susceptibility to react favorably or unfavorably to something or someone [36]. It describes how you feel about an object, just like how you feel about someone or anything when you like or dislike them. Hence, a person’s attitude toward exhibiting a behavior is determined by whether they think it is favorable or negative. Individuals’ overall propensity to engage in or refrain from engaging in behavior can be predicted in part by their attitude. It also explains why people judge behavior to be good or negative [37]. It is crucial to realize that attitude, in contrast to a value, cannot be seen or touched because it is an abstract concept. As opposed to this, attitude can be understood from what people say or do [36]. Attitude shapes the behavior of an individual by filtering information and developing perceptions about the surroundings. Researchers [38] attempted to expand the TAM by considering both cognitive and affective components of attitude to explain information system use. However, the study found that only cognitive attitude acted as an essential factor in justifying the use of information systems. Recent studies in the context of e-learning found attitude a vital predictor in explaining the intention to use e-learning [39,40,41,42].

#### 1.1.5. Hypotheses Development

Previous studies found that the level of social influence exerted by supervisors and peer groups and the social presence of the medium influence the perceived usefulness of technology among individuals. Further, an individual’s perception of the ease of use of technology is stemmed from her/his self-efficacy with the technology [35,43]. Self-efficacy acts as a significant antecedent of PU and PEU of technology. Self-efficacy plays an essential role in shaping the beliefs and behavior of individuals toward the use of technology [44,45]. However, self-efficacy can only be achieved by physical access to the system. Researchers [46,47] argued that extensive support and training on the newly introduced system and ready physical access to the system would facilitate the acceptance of the new system among individuals. In view of the above-mentioned argument, it can be claimed that external factors (self-efficacy, system accessibility, and subjective norms influence PU and PEU. Therefore, we hypothesize the following:

**H1.**  *Self-efficacy has a constructive influence on the perceived usefulness of e-learning among academicians.*

**H2.**  *Self-efficacy has a positive influence on the perceived ease of use of e-learning among academicians.*

**H3.**  *System accessibility has a positive influence on the perceived usefulness of e-learning among academicians.*

**H4.**  *System accessibility has a positive influence on the perceived ease of use of e-learning among academicians.*

**H5.**  *Subjective norm has a positive influence on the perceived usefulness of e-learning among academicians.*

**H6.**  *Subjective norm has a positive influence on the perceived ease of use of e-learning among academicians.*

Recent studies on e-learning have found that PU, PUE, and attitudes are strong predictors of using technology and the intention to use it [39,40,41,42]. Park [48] found no direct relationship between perceived ease of use, perceived usefulness, and intention to use e-learning among university students. However, he found that perceived ease of use and perceived usefulness had a proportionate relationship with attitude towards the use of e-learning, which eventually led to the intention to use e-learning. Ansong-Gyimah [3] found that attitude towards the use of e-learning mediated the relationship between perceived ease of use, perceived usefulness, and intention to use e-learning. Studies found that attitude towards the use of e-learning was one of the most considerable predictors of e-learning use [39,41]. Another study found that PU and PEU were non-significant variables in predicting intention to use [41]. The findings of recent studies establish that attitude towards use is an essential component of TAM. It strongly predicts the intention to use e-learning.

The research findings of previous studies performed in non-educational settings also establish the relationship between PU, PEU, attitude towards the use of technology, and intention to use [49,50,51]. Guritno and Siringoringo [52] found that the variable which had a highly considerable impact on attitudes toward the usability of online tickets was perceived usefulness. They argued that when consumers perceive high benefits in using technology, they have a constructive attitude towards using the technology. Seyal and Rahman [53] also found that perceived usefulness had the most significant influence on the intention to use the Internet among university students. In a study, it was found that perceived usefulness, ease of use, security, and privacy were significant antecedents of customer attitudes toward using internet banking. A study performed in the context of crisis response strategy found a significant mediating role of online brand attitude and online purchase intention [54,55]. The study also found that brand-perceived usefulness significantly strengthens the positive relationship between online brand attitude and online purchase intention. The above-mentioned studies provide enough support to hypothesize that.

**H7.**  *Perceived usefulness has a positive influence on attitudes toward e-learning among academicians.*

**H8.**  *Perceived ease of use has a positive influence on attitudes toward e-learning among academicians.*

**H9.**  *Attitude toward e-learning has a positive influence on the intention to use e-learning among academicians.*

## 2. Materials and Methods

### 2.1. Data Collection Procedure and Sampling

**Figure 1:**
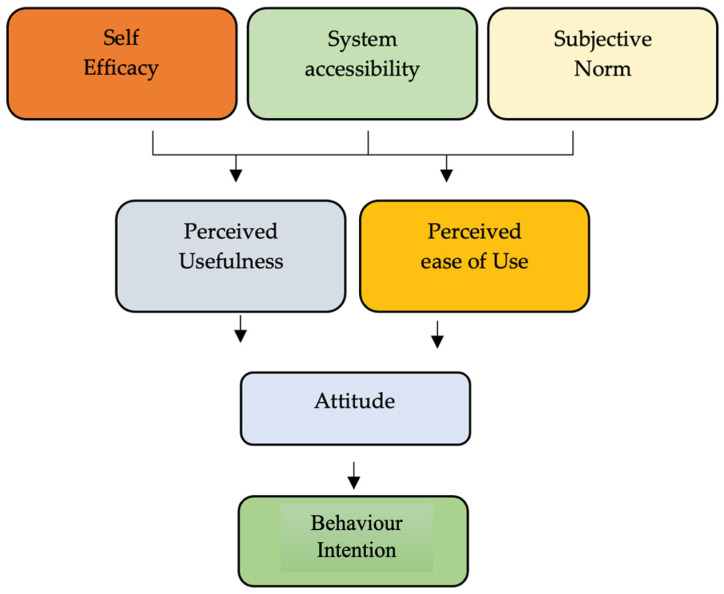
Research Framework.

The study’s objective is to explore academicians’ behavioral intention to use e-learning during the COVID-19 crisis in the Kingdom of Saudi Arabia. A cross-sectional quantitative survey was conducted using a structured questionnaire during the period from December 2020 to October 2021 to address the objectives. The non-probability purposive sampling technique was used to collect the data from a target of 300 respondents. However, at the end of the data collection process, 263 valid questionnaires were considered in the final analysis, and the response rate was 87.66%. The respondents consisted of academicians working in higher education institutions inside the Kingdom of Saudi Arabia during the COVID-19 crisis. The questionnaire was carefully designed and developed to address the objectives of the study. The questionnaire consists of close-ended responses such as socio-demographic information, variables such as self-efficacy, system accessibility, subjective norms, perceived usefulness, perceived ease of use, attitude and behavioral intention (Figure 1). (23 items using a 5-point Likert scale, with scores ranging from 1 = Strongly Agree to 5 = Strongly Disagree) and (d) open-ended questions for opinions on improving e-learning among academicians.

Ethical approval for the data collection of the research study was obtained from the Ethical Committee of Saudi Electronic University (SEUEC/File no./4239-14 October 2020). Written informed consent was obtained from the participants before starting the survey, and a detailed explanation was provided to participants when needed. The responses were carefully captured and coded in SPSS 21.0 statistical package software for analysis. Cronbach’s alpha is used for measuring the internal consistency, i.e., the validity and reliability of the data, and found Cronbach’s alpha is 0.84.

Sekaran et al. [56] described “purposive sampling the study subjects who can provide the required information, ones who have it or conform to some criteria set by the researcher”. Informed consent was obtained after explaining the objectives to the respondents of the study. Harman’s single-factor test was performed to identify the common method bias in the survey data. The minimum sample size was estimated using structural equation modelling, Chin et al. [57] (2010a) elucidated that the sample size should be equal to or 10 times greater than the number of structural paths pointing to an estimated minimum sample size. Further, as per the study published by Reinartz, Haenlein, and Henseler [58], the sample size of 100 is acceptable for a study conducted with partial least square-structural equational modeling (PLS-SEM). Therefore, the estimated sample size of 263 was adequate to operate the PLS-SEM analysis.

### 2.2. Measurements

The study tool was developed using well-tested scales, and items were adapted from earlier studies. The items for constructing self-system accessibility (3 items), efficacy (4 items), and subjective norms (3 items) were adapted from Sung Youl Park et al. [59]. Similarly, items for the constructs perceived usefulness (4 items), attitude (4 items), perceived ease of use (4 items), and behavioral intention (3 items) were extracted from the original TAM [22]. The items were measured on a five-point Likert scale that ranged from (1) strongly disagree to (5) strongly agree.

## 3. Results

The respondents of the study were academicians from institutions of higher education in Saudi Arabia. Non-Saudi residents were the predominant respondents. Male respondents were (63%) exceeding female respondents. The majorities of the participants were (56%) 36–45 years age group. Many of the respondents hold Ph.D. degrees (61%) and have job experience of 6–10 years (46%). Table 1 details the profile of the respondents.

### 3.1. Assessment of Measurement Model

The assessment is done through factor loading, average variance extracted (AVE), and composite reliability (CR) to check the validity of the research model. The ceiling value for the factor loading, AVE, and CR are 0.708, 0.7, and 0.5, respectively, as per the recommendation [60]. The mean, standard deviation (SD), factor loadings, AVE, and CR of latent variables of all the items are analyzed in Table 2 below. As per the analysis, only PEU3 (0.524) was lower than the recommended value of 0.708. The validity of the research model is determined with the help of the Heterotrait–Monotrait (HTMT) ratio as it is a more powerful criterion compared to Fornell–Larcker method [61]. The discriminant validity of the study model was established by using the ceiling value of 0.90 for HTMT, in which all the values are below 0.90, as discussed in Table 3 below.

### 3.2. Assessment of Structural Model

The significance of the path coefficient (β-value) and the coefficient of variance (R^2^) is used for the structural model in PLS-SEM [62]. As per Cohen (1988), R^2^ values 0.02–0.12, 0.13–0.25, and 0.26 and above are considered weak, moderate, and substantial, respectively. However, Hair et al. [62] (2011) qualified these figures and suggested that high R^2^ is dependent on a specific research context. The Table 4 analysis suggests that the R^2^ values of perceived usefulness and perceived ease of use were 0.035 and 0.0215, respectively. The R^2^ value of the construct attitude was 0.169, and the R^2^ value of intention to use was 0.069. For assessing the PLS-SEM, the path coefficient is used. This path coefficient was determined by comparing the t-values to the critical t-values for significance levels of 0.05 (one tail), and for calculating the t-value, Bootstrapping was used for 1000 subsamples as recommended by Hair Jr et al. [63].

The data analysis results showed that self-efficacy was positively related to perceived usefulness at β = 0.143 and *p* < 0.05; therefore, H1 was found supported, but H2 was found not supported as self-efficacy had no relationship with perceived ease of use at β = 0.057 at *p* > 0.05. The system accessibility had a significant and positive relationship with perceived usefulness at β = 0.283 and *p* < 0.01 supporting H3 and a significant positive relationship with perceived ease of use at β = 0.247 and *p* < 0.01 supporting H4. There is no subjective norm relationship with perceived usefulness at β = −0.065 at *p* > 0.05 and perceived ease of use at β = −0.012 at *p* > 0.05, making H5 and H6 unsupported, respectively. The perceived usefulness had a positive relationship with attitude at β = 0.168 and *p* < 0.05; perceived ease of use had a significant positive relationship with attitude at β = 0.556 and *p* < 0.01, and attitude had a significant positive relationship with intention to use at β = 0.262 and *p* < 0.01, supporting H7, H8, and H9 respectively.

The predictive accuracy of the model was assessed by using Q^2^ along with Stone-Geisser’s Q^2^ value to determine the model’s predictive relevance. The indication for predicting the data points of the endogenous constructs is when the Q^2^ value is greater than zero [58]. For obtaining the Q^2^ value, blindfolding in Smart-PLS was performed by omitting every sixth data point in the endogenous construct indicators and using construct cross-validated redundancy. These omitted data points were then treated as missing data in Smart-PLS, and the difference between the omitted data points and the predicted ones is used for calculating the Q^2^ [58]. The Stone-Geisser’s Q^2^ values for the endogenous constructs of the study model are 0.365, 0.318, 0.546, 0.219 for the perceived usefulness, perceived ease of use, attitude, and intention to use.

## 4. Discussion

Change is inevitable, and COVID-19 has brought changes in all facets of life, including the education sector, and made it mandatory to adopt a distance learning model. This study aimed to investigate the consequence of external factors on the perceived usefulness and perceived ease of use of e-learning and the further outcome of these perceptions on attitude and intent to use e-learning among academicians at higher education institutions in Saudi Arabia using TAM. One of the e-learning acceptance models used to recognize and improve staff and student attitudes is the TAM [23]. This model also states that when individual views such as perceived ease of use and perceived usefulness of new technology are high, persons would possess a constructive attitude toward new technology that creates an intention to use the technology. According to the TAM hypothesis, a person’s intention to use technology and their behavior when using it depends on how convenient and beneficial they perceive technology to be [24]. In the present study, Tam was used to determine the effects of external factors on the perceived ease of use and perceived usefulness of e-learning and the further effect of these perceptions on attitude and intention to use e-learning by academicians.

When referring to a person’s perception of her or his ability to complete a task or engage in an activity, self-efficacy plays a crucial role in the development of motivation and behavior [27,28]. “Self-efficacy” is the belief in one’s own skills to use cognitive resources. On the other hand, the subjective norm refers to the degree to which a person takes into account how others may affect his or her actions. Perceived usefulness is the idea that a person’s job performance has improved proportionately to the use of technology. In contrast, perceived ease of use reflects how much effort a person puts into using the technology. Attitude is the propensity to react favorably or unfavorably to something or someone [36]. It describes how you feel about an object, just like how you feel about someone or anything when you like or dislike them. In the present study, the following parameters were assessed in the academicians of higher education institutes, which helped in determining the perceptions, attitudes, and intentions of the application of e-learning among academicians during the crisis.

This study was developed to connect the research gap by investigating the association among the variables of the technology acceptance model (TAM) in the framework of the sudden change from traditional classroom teaching to virtual teaching in Saudi higher education institutions due to the COVID-19 outbreak. Though earlier studies have explored the relationship among the factors explained in the TAM during normal environmental conditions where changes in methods of learning were brought gradually and systematically, this study focused on the COVID-19 outbreak.

The conclusions of the study discovered that self-efficacy and system accessibility have a significant influence on the perceived usefulness of e-learning, and system accessibility has a significant influence on the perceived ease of use of e-learning, similar to the previous studies [22,35]. However, this study found that self-efficacy had no relationship to perceived ease of use, which was contrary to the findings of previous studies [43,45,64,65,66], pointing out the reason being the sudden adoption of e-learning in higher education institutions due to the COVID-19 outbreak. As the outbreak of COVID-19 was rapid and large-scale, institutions had no choice but to transfer the teaching from classroom teaching to virtual rapidly. Due to this, academicians also might not have the opportunity and freedom to ponder on the feasibility and ease of use of e-learning methods as they have swiftly adopted and delivered virtual teaching. Even though there was no scope for long and exhaustive training for the academicians, they could have developed self-efficacy, which leads to an ease of use of e-learning.

This study hypothesized the positive relationship of the subjective norm with perceived usefulness and perceived ease of use. However, the findings of the study revealed that there was no relationship between subjective norm and perceived usefulness and perceived ease of use. This is an interesting finding as previous studies investigated the relationship between subjective norm and perceived usefulness and perceived ease of use in the context of the adoption of e-learning among academicians are scarce. Previous studies have investigated the relationship between these factors in the context of the use of social media, e-portfolio, and internet banking among students and found a significant relationship between these variables [67,68]. However, this study found that subjective norm has no role in developing a perception of the usefulness and ease of use of e-learning among academicians.

This study also found a significant relationship between perceived usefulness, perceived ease of use, and attitude toward adopting e-learning among academicians. These findings are similar to the previous studies; however, few studies found no relationship between perceived ease of use and attitude towards the use of technology [69,70,71,72]. The study also found a positive and significant relationship between attitudes toward using e-learning in changed scenarios due to the COVID-19 outbreak and the intention to use it among academicians. This finding was also similar to the previous study findings performed in the context of the use of technology [32,73].

### Theoretical and Practical Implication

The outcomes of the research study offer both theoretical and practical implications of interest to academicians, institutes of higher education, and policymakers. The verdicts of the study confirm the usability of the technology acceptance model (TAM) in understanding the intention of the use of e-learning among academicians in a situation of crisis. The present study confirms that TAM still has relevance in forecasting the behavior intention of individuals in adopting the technology. Furthermore, the findings of the research study establish that attitude has a vital role in predicting the technology use intention of individuals, which is contrary to the few previous studies that have written off the role of attitude in TAM [74,75]. The research study conceptualizes the extension of TAM by testing the properties of external factors on perceived usefulness and perceived ease of use. The three external factors were self-efficacy, system accessibility, and subjective norm. There were very few studies that used system accessibility as a predictor to explain the perceived usefulness and perceived ease of use in the context of the goal to use technology. The present study found that system accessibility has a significant and positive influence on perceived usefulness and perceived ease of use.

Another significant theoretical implication of the present study is the use of TAM in explaining the intended behavior of academicians to use e-learning. Many previous studies have used TAM to measure the intention of students to use e-learning methods such as social media, m-learning, and Internet learning [41,76,77]. However, studies lack the use of TAM to explore the behavioral intentions of academicians. The findings of this study establish the usefulness of TAM in academic settings. The study found that perceived usefulness and perceived ease of use were strong predictors of attitude to use e-learning. This means it is important that academicians must be elaborated precisely on the job benefits of using e-learning, especially in crises. Perceived performance benefits will lead to developing attitudes among academicians to use e-learning. Similarly, exhaustive training is important to develop confidence among academicians so that they can smoothly operate the new technology. That will also lead to a developing attitude among academicians to decently use e-learning [78]. The study uses three external factors as predictors of perceived usefulness and perceived ease of use. The findings of the study detailed that self-efficacy has a positive influence on perceived usefulness. Self-efficacy refers to an individual’s perception of her/his ability to perform a task or engage in an activity [27,28,79,80,81,82]. Bandura [82], in his seminal work, explained that self-efficacy could be developed by exposing herself/himself to the task, witnessing others complete the task, the self-belief that she/he is capable of taking the task, and the emotional state of the individual. Academicians must keep themselves exposed to information technology and develop at least a medium level of operating capability of information technology so that they can easily switch to e-learning if there is an urgent need. Basic knowledge of information technology will put academicians in a position where they can exploit the benefits of high job performance by using e-learning. The study findings revealed that self-efficacy had no relationship with perceived ease of use, which is fairly understood. If an individual possesses self-efficacy in information technology, she/he will not be distressed about the ease of use of new technology, which further strengthens the view that it is a must for individuals in the era of information technology to develop self-efficacy in it [78,79,80,81,82].

The study found that system accessibility has a positive influence on perceived usefulness and perceived ease of use. System accessibility means physical access to information technology machines, in other words, a computer system. Academicians should be provided with quality/latest machines so that they can develop command and capability on the machine and perform their tasks effectively. The study also found that subjective norms had no relationship with perceived usefulness and perceived ease of use. In organizational settings, subjective norms mean the opinion of the supervisor and peer group. However, in higher education settings, academicians do not operate in groups and work individually and independently to perform their job-related tasks. Therefore, it is obvious that academicians would be unaffected by the opinion of peer groups about their efficiency in the use of e-learn. Lastly, the management of institutions of higher education should find ways to keep academicians informed about the contemporary information technology being used in e-learning by for exampling organizing seminars and sessions to keep academicians informed about the technology in e-learning. Further, policymakers should develop policies that encourage institutes of higher education to implement e-learning gradually and steadily.

## 5. Conclusions

The research study was conceptualized to investigate the influence of external factors (self-efficacy, system accessibility, and subjective norm) on perceived usefulness and perceived ease of use, which further influence attitudes and intention to use e-learning among academicians of higher education institutions through the time of the crisis (COVID-19 outbreak). The research study has novelty as not many previous studies used TAM to measure the intention to use e-learning during a crisis time. Further, till now, the TAM has not been extensively tested in the context of the intention to use e-learning among academicians in higher education institutions. The findings of the study disclosed that perceived ease of use was the most important factor (β = 0.556) in developing the attitude among academicians to use e-learning, followed by perceived usefulness (β = 0.262). Furthermore, it can be concluded that system accessibility has a stronger influence on developing perception among academicians about the usefulness and ease of use of e-learning than self-efficacy. The limitations of the study included the use of non-probability purposive sampling to collect the data though the sample size of 263 could be adequate for the study’s framework when inspected on G*power in a priori power analysis [76]. Further, the representation of female respondents was comparatively less (37%). Future studies should use the equal distribution of male and female representation in the study sample to further increase the generalizability of the study findings. The study bridges the research gap by applying the TAM on academicians of higher education institutions to measure the intention to use e-learning during the time of the crisis. Future studies are recommended to analyze the effect of the use of learning on the behavior knowledge and development of skills in students and also the perception of instructors for it.

## Figures and Tables

**Table 1:** Socio-demographic profile of the respondents.

Categories	Category	Number	Percent (%)
Nationality	Non-Saudi	200	76
	Saudi	63	24
Gender	Male	166	63
	Female	97	37
Age group (Years)	26–35	83	31
	36–45	146	56
	46–55	34	13
Education	Ph.D.	161	61
	Masters	102	39
Experience (years)	0–5	58	22
	6–10	122	46
	11–20	78	30
	21 and more	5	2

**Table 2:** Results of the Measurement Model.

Latent Variable	Mean	SD	Factor Loading	CR	AVE
Self-efficacy (SE)	3.743	0.728		0.925	0.861
SE1			0.912		
SE2			0.836		
SE3			0.924		
SE4			0.823		
System accessibility (SA)	3.768	0.757		0.921	0.754
SA1			0.879		
SA2			0.970		
SA3			0.838		
Subjective norms (SN)	3.938	0.787		0.948	0.82
SN1			0.921		
SN2			0.933		
SN3			0.827		
Perceived usefulness (PU)	3.773	0.673		0.933	0.776
PU1			0.902		
PU2			0.839		
PU3			0.843		
PU4			0.857		
Perceived ease of use (PEU)	3.564	0.535		0.868	0.628
PEU1			0.867		
PEU2			0.896		
PEU3			0.524		
Attitude (AT)	3.238	0.578		0.871	0.628
AT1			0.883		
AT2			0.796		
AT3			0.861		
Behavioural Intention (BI)	3.978	0.643		0.856	0.754
BI1			0.865		
BI2			0.886		
BI3			0.754		

**Table 3:** Discriminant Validity (HTMT_0.90_).

		1	2	3	4	5	6	7
1	Self-efficacy							
2	System accessibility	0.63						
3	Subjective norms	0.603	0.539					
4	Perceived usefulness	0.748	0.665	0.663				
5	Perceived ease of use	0.502	0.548	0.73	0.678			
6	Attitude	0.869	0.611	0.683	0.826	0.676		
7	Behavioural Intention	0.593	0.784	0.448	0.689	0.778	0.638	

**Table 4:** Structural Model Analysis.

Hypo Thesis	Relationship	Beta	SE	T-Value	*p*-Value	Decision
H1	Self-efficacy -> Perceived usefulness	0.143	0.086	1.654	0.048	Supported
H2	Self-efficacy -> Perceived ease of use	0.057	0.093	0.631	0.266	Not supported
H3	System accessibility -> Perceived usefulness	0.283	0.087	3.215	0.001	Supported
H4	System accessibility -> Perceived ease of use	0.247	0.087	2.804	0.004	Supported
H5	Subjective Norms -> Perceived usefulness	−0.065	0.087	0.723	0.234	Not supported
H6	Subjective Norms -> Perceived ease of use	−0.012	0.094	0.124	0.452	Not supported
H7	Perceived usefulness -> Attitude	0.168	0.081	2.058	0.030	Supported
H8	Perceived ease of use -> Attitude	0.556	0.067	8.104	0.000	Supported
H9	Attitude -> Intention to use	0.262	0.085	3.024	0.001	Supported

## Data Availability

Data can be made available on demand by the chief researcher for academic purposes by email.
