# Correction: Khan et al. Challenges of E-Learning: Behavioral Intention of Academicians to Use E-Learning During COVID-19 Crisis. *J. Pers. Med.* 2023, *13*, 555

**DOI:** 10.3390/jpm16020114

**Published:** 2026-02-14

**Authors:** Mohammad Jamal Khan, Lingala Kalyan Viswanath Reddy, Javed Khan, Bayapa Reddy Narapureddy, Sunil Kumar Vaddamanu, Fahad Hussain Alhamoudi, Rajesh Vyas, Vishwanath Gurumurthy, Abdelrhman Ahmed Galaleldin Altijani, Saurabh Chaturvedi

**Affiliations:** 1 Department of Public Health, College of Health Sciences, Saudi Electronic University, Riyadh 13316, Saudi Arabia; 2 Department of Public Health, College of Health Sciences, Saudi Electronic University, Abha 61421, Saudi Arabia; 3 Department of Public Health, College of Applied Medical Sciences, Khamis Mushayt, King Khalid University, Abha 61421, Saudi Arabia; 4 Department of Dental Technology, College of Applied Medical Sciences, King Khalid University, Abha 61421, Saudi Arabia; 5 Department of Prosthetic Dentistry, College of Dentistry, King Khalid University, Abha 61421, Saudi Arabia

**References**

The references of the published paper [1] have been corrected. References numbered 37 and 54 have been replaced to the following references:

37. Mahyoob, M. Challenges of e-Learning during the COVID-19 Pandemic Experienced by EFL Learners. *Arab World Eng. J.* **2020**, *11*, 4.

54. Abdur Rehman, M.; Soroya, S.H.; Abbas, Z.; Mirza, F.; Mahmood, K. Understanding the challenges of e-learning during the global pandemic emergency: The students’ perspective. *Qual. Assur. Educ.* **2021**, *29*, 259–276.

References numbered 55–58, 81–83, 85–88, 90–92 and 95 have been removed.

With this correction, the order of some references has been adjusted accordingly. The authors state that the scientific conclusions are unaffected. This correction was approved by the Academic Editor. The original publication has also been updated.
